# Contributions of emotional state and attention to the processing of syntactic agreement errors: evidence from P600

**DOI:** 10.3389/fpsyg.2015.00388

**Published:** 2015-04-09

**Authors:** Martine W. F. T. Verhees, Dorothee J. Chwilla, Johanne Tromp, Constance T. W. M. Vissers

**Affiliations:** ^1^Centre for Cognition, Donders Institute for Brain, Cognition and Behaviour, Radboud University, NijmegenNetherlands; ^2^Max Planck Institute for Psycholinguistics, NijmegenNetherlands; ^3^Kentalis Academy, Sint-MichielsgestelNetherlands

**Keywords:** emotion, mood, syntactic processing, P600, attention, levels of processing

## Abstract

The classic account of language is that language processing occurs in isolation from other cognitive systems, like perception, motor action, and emotion. The central theme of this paper is the relationship between a participant’s emotional state and language comprehension. Does emotional context affect how we process neutral words? Recent studies showed that processing of word meaning – traditionally conceived as an automatic process – is affected by emotional state. The influence of emotional state on syntactic processing is less clear. One study reported a mood-related P600 modulation, while another study did not observe an effect of mood on syntactic processing. The goals of this study were: First, to clarify whether and if so how mood affects syntactic processing. Second, to shed light on the underlying mechanisms by separating possible effects of mood from those of attention on syntactic processing. Event-related potentials (ERPs) were recorded while participants read syntactically correct or incorrect sentences. Mood (happy vs. sad) was manipulated by presenting film clips. Attention was manipulated by directing attention to syntactic features vs. physical features. The mood induction was effective. Interactions between mood, attention and syntactic correctness were obtained, showing that mood and attention modulated P600. The mood manipulation led to a reduction in P600 for sad as compared to happy mood when attention was directed at syntactic features. The attention manipulation led to a reduction in P600 when attention was directed at physical features compared to syntactic features for happy mood. From this we draw two conclusions: First, emotional state does affect syntactic processing. We propose mood-related differences in the reliance on heuristics as the underlying mechanism. Second, attention can contribute to emotion-related ERP effects in syntactic language processing. Therefore, future studies on the relation between language and emotion will have to control for effects of attention.

## Introduction

Emotions have an influence on how we see the world. Consider the emotion fear. Fear spreads through the human body and brain and it urges us to take actions for flight or defense. Furthermore, fear directs our attention to signs of danger or safety in the environment. The brain has, so to speak, been simplified to respond to danger and this mode is turned on by the emotional signal. The emotions we experience thus color the way we perceive the world around us. The aim of the present study was to investigate the relationship between emotional state, attention and language processing.

Emotional state, or mood, refers to a generalized affective state that is not directed at objects or events and has been proposed to be less intense and longer-lasting than emotions (Frijda, 1986; Morris, 1989; Isen, 1993; Watson and Clark, 1994; Oatley et al., 2006). Emotional state has been shown to influence perception, thinking and decision making, and the notion of mood-dependent processing styles is generally agreed upon in the emotion literature (Levenson, 1994; Fredrickson and Branigan, 2005). Positive mood is associated with flexibility of thinking and with a more global, category level information processing style. People in a good mood use heuristics (highly economical perceptual strategies), rely on their world knowledge and on their usual routines (e.g., Isen, 2001; Gasper and Clore, 2002). Negative mood, on the other hand, is associated with a more local, bottom–up, analytic and systematic information processing style, in which people rely less on heuristics and have a narrowed focus of attention (Gasper and Clore, 2002; Schwarz, 2002).

Attention is a processing system with limited capacity that can allocate its resources flexibly to one or more tasks (Kahneman, 1973). Several studies have shown that emotion influences attentional processing (e.g., Vuilleumier, 2005; see for an overview: Kaspar and König, 2012). For example, the internal state of a person (including emotional state) has been found to have an influence on visual attention (Kaspar and König, 2012). Less investigated, however, is whether attention also has an influence on emotion.

Relevant for the present study, recent fMRI studies have shown that emotion and cognition (memory, attention, language) strongly interact in the brain and that the neural basis of emotion and cognition should be viewed as interactive and non-modular (e.g., Pessoa, 2008). Likewise, research of language in interaction with other systems has revealed that processes of language comprehension do not operate in isolation but are affected by perception and action (see for embodied approaches to cognition, e.g., Glenberg, 1997; Barsalou, 1999; Pecher and Zwaan, 2005 and for event-related potential (ERP) support, e.g., Chwilla et al., 2007; Chwilla, 2013). More recently the relationship between emotion and language processing has been examined and interactions between language and a person’s emotional state have been reported (e.g., Federmeier et al., 2001; Vissers et al., 2010; Chwilla et al., 2011; Pinheiro et al., 2013; Van Berkum et al., 2013). These interactions of language with other systems are of theoretical importance because they call into question modular theories of language and support interactive theories of language.

The focus of this article is on the relative contribution of emotional state and attention on the processing of syntactic anomalies. The ERP method is used to track effects of emotional state and attention on syntactic language processing online. An advantage of ERPs is that they have an excellent temporal resolution at the level of milliseconds, which allows to assess the effects of emotion and attention on language processing in real time. To our knowledge, the relationship between emotional state, attention and syntactic language processing has not yet been investigated.

Two for the present study language-relevant ERP components are the N400 and the P600. The N400 is a negative-going brain wave peaking around 400 ms after critical word onset and is highly sensitive to semantic processing (see for a review: Kutas and Federmeier, 2011). The P600 component is a positive shift peaking at around 600 ms after critical word onset. P600 has been shown to be sensitive to several syntactic anomalies and ambiguities (see e.g., Osterhout and Holcomb, 1992; Coulson et al., 1998a and for a review: Kutas et al., 2006). For instance, an increase in P600 amplitude has been observed after subject–verb agreement violations (e.g., Hagoort et al., 1993) and after phrase structure violations (e.g., Hahne and Friederici, 1999). More recently, it has been shown that a P600 can also occur to semantic anomalies in syntactically unambiguous sentences (see for reviews: Kuperberg, 2007; Van de Meerendonk et al., 2009). The focus of the current study is on the effect of syntactic violations, specifically subject–verb agreement errors, on P600. The typical finding is that P600 amplitude to syntactically incorrect words is larger (i.e., more positive going) than to syntactically correct words. This difference in P600 amplitude to incorrect vs. correct sentences is referred to as the P600 effect (Osterhout and Holcomb, 1992).

Event-related potential studies concerning the relationship between emotional state and language processing have consistently shown that emotional state influences semantic processing. In particular, effects of mood have been found on the use of semantic memory during reading (Federmeier et al., 2001; Pinheiro et al., 2013), on the standard N400 cloze probability effect (Chwilla et al., 2011) and on semantic integration in discourse comprehension (Egidi and Nusbaum, 2012).

Whether emotional state impacts syntactic language processing is a matter of debate. Given that studies on the effect of mood on syntactic processing are of direct relevance for the present article, they will be presented in more detail. In one study, a mood-related modulation of the P600 effect was found (Vissers et al., 2010). The authors manipulated mood (happy vs. sad) by showing affective film clips and studied its effect on the processing of emotionally neutral sentences with or without subject–verb agreement. Vissers et al. (2010) reported a broadly distributed P600 effect for the happy mood condition and a strong reduction in P600 effect for the sad mood condition. They propose three possible scenarios to account for the effect of emotional state on P600. The first scenario is that the mood by syntactic correctness interaction could be accounted for in terms of syntactic factors. That is, mood could affect language comprehension by increasing or decreasing syntactic processing. A second scenario is that mood could affect language processing via more general factors such as attention. On this account people in a happy mood might pay more attention to the sentences than people in a sad mood. According to a third scenario, mood selectively affects the use of heuristics. Heuristics are highly economical perceptual strategies that are usually very effective in extracting meaning and allow people to quickly solve problems and make judgments (e.g., Ferreira, 2003). On the assumption that people have a high expectancy for sentences to be syntactically correct (e.g., Coulson et al., 1998a; Vissers et al., 2013), the reduction of the P600 effect in the sad mood condition could reflect a reduced use of heuristics, whereas the increase in P600 effect for the happy mood could be due to an increased reliance on heuristics.

In contrast, in a recent study using a similar procedure to induce a happy vs. sad mood and the same type of syntactic anomaly (subject–verb agreement errors) no modulation of P600 by mood was obtained (Van Berkum et al., 2013). Note that Van Berkum et al. (2013) did observe a standard P600 effect to syntactic anomalies across mood conditions. The only difference between moods consisted of a slightly earlier onset of the P600 effect in the happy mood condition as compared to the sad mood condition. From this the authors conclude that emotional state has little impact on syntactic processing. In a third study by Jiménez-Ortega et al. (2012) mood was manipulated by presenting participants with emotionally positive, negative or neutral text paragraphs preceding a critical sentence that contained a syntactic anomaly (noun-adjective number disagreements). While these researchers did find an effect of emotional state on behavioral measures (i.e., higher error rates and reaction times (RTs) in happy mood compared to sad mood), no modulation of P600 by emotional state was observed. The authors explain the absence of a mood by syntactic correctness interaction in their study vs. presence of such an interaction in the Vissers et al. (2010) study in terms of the effectiveness of the mood induction procedure. Specifically, they propose that the emotional paragraphs might not have been effective in inducing the intended emotional state. This would also explain why this is the only ERP study in which no effect of emotional state on semantic processing – as reflected by N400 – was obtained.

Of interest for the present purposes there was one study that looked at the influence of depression on syntactic processing as indexed by P600 (Ruchsow et al., 2008). Patients with depression and healthy controls were presented with sentences containing syntactic mismatches. The authors found that, in contrast to healthy controls, patients with depression did not show a significant P600 effect to syntactic anomalies. Ruchsow et al. (2008) conclude that the absence of P600 effect in depressed patients points to an altered syntactic integration process. They state that it is an open question whether this altered syntactic integration process indicates a specific language processing deficit or is part of a more general cognitive deficit. If normal sadness and depression reflect a qualitatively similar process, then based on this finding one would predict a reduction in P600 effect for sad mood.

It has been proposed that an effect of emotional state on language processing can be influenced by more general factors like attention (Vissers et al., 2010, 2013; Chwilla et al., 2011; Van Berkum et al., 2013). Previous ERP studies investigating the effects of attention on syntactic processing have found that the P600 is modulated by list composition (a high vs. low proportion of syntactically correct vs. incorrect sentences), which induces differences in expectancy for correct or incorrect sentences (e.g., Coulson et al., 1998a). Relevant for the present article, Gunter and Friederici (1999) showed that depth of processing modulates the amplitude of the P600. They used a deep processing task and a shallow processing task to induce different levels of processing. In the deep processing task, participants had to judge whether a sentence was grammatically correct or not. In the shallow processing task, participants had to judge the sentences on purely physical features, i.e., whether a sentence contained a word in a deviant letter size. This task manipulation modulated P600 amplitude: in the physical judgment task a strong reduction of the P600 effect following incorrect verb inflections was observed as compared to the syntactic judgment task (see for a similar task modulation of N400: Chwilla et al., 1995).

The goal of the present study was twofold. First, to clarify whether and if so how emotional state affects syntactic processing. If syntactic processing is reliably affected by mood, this would further challenge modular views of language comprehension according to which syntactic processing is encapsulated (see e.g., Fodor, 1983). The second goal is to shed light on the underlying mechanisms by separating possible effects of mood on syntactic processing from those of attention. The crucial question is whether the effects of emotion and attention on P600 are additive and independent or whether they interact. In other words, if emotional state modulates P600, is this modulation then a true effect of emotion or could it be influenced by more general factors like attention?

To the first aim, we induced a happy mood or sad mood and presented sentences with or without subject–verb agreement errors to participants while their EEG was recorded. It has been well-established that subject–verb agreement errors elicit a P600 effect (see for a review: Vos et al., 2001). Emotional state (happy vs. sad) was manipulated by presenting film clips. It has been shown that the presentation of a film or story with explicit instructions to enter a specific mood is one of the most effective ways to induce both positive and negative emotional states (Westermann et al., 1996). For the happy mood induction, film clips from a happy movie, Warner Brother’s “Happy Feet” were used. For the sad mood induction, fragments from a sad movie, “Sophie’s choice” were used. Fragments from the same movies successfully induced the intended mood in previous studies (Vissers et al., 2010, 2013; Chwilla et al., 2011).

To the second aim, we manipulated attention in addition to the factor mood. Attention was manipulated in the same way as in the Gunter and Friederici (1999) study, by directing attention to syntactic features vs. physical features of the sentences. Specifically, participants either had to indicate whether the sentence was syntactically correct or whether the sentence contained a word in a deviant letter size. In the present study the factors emotional state (happy vs. sad) and task (syntactic vs. physical) are crossed. This design allows a determination of the relative role of the factors mood and attention (varied by task demands) in the processing of syntactic anomalies.

Based on previous ERP studies investigating the effects of mood and attention on the processing of syntactic anomalies, the predictions were as follows: first of all, we predicted a standard P600 effect to syntactic anomalies across mood and task conditions. As aforementioned, it is a matter of debate whether emotional state modulates the P600 effect to syntactic violations. If mood affects the size of the P600 effect we predict an interaction between mood and syntactic correctness, in particular a reduction in P600 effect for the sad mood as compared to the happy mood condition (Vissers et al., 2010). In contrast, if P600 is mainly insensitive to fluctuations in emotional state then no differences in P600 amplitude as a function of mood should be obtained (Jiménez-Ortega et al., 2012; Van Berkum et al., 2013).

Manipulation of the factor attention alongside the factor mood makes it possible to assess the (relative) contribution of attention to a mood-related modulation of P600. If general factors like attention contribute to an effect of emotional state on P600, this should be reflected in an interaction between emotional state, task and the P600 effect of syntactic correctness. On the other hand, if attention does not contribute to an effect of emotional state on P600, no interaction between emotional state, task and the P600 effect should be obtained. In the latter case an effect of emotional state and/or an effect of attention should be found on P600, in the absence of an interaction.

## Materials and Methods

### Participants

There were 38 participants (mean age = 20 years, age range = 18–26). Recent research has shown that the assumption that subject sex matters little or not at all in studies on the neurobiology of emotional memory should be abandoned (Cahill, 2006). In line with this, a previous ERP study suggested that female participants are more sensitive to mood manipulations (Federmeier et al., 2001). Therefore, only female participants were tested in this study. Furthermore, only participants that reported no drug abuse, neurological, mental or chronic bodily diseases, or medication for any of these were selected. All participants were native speakers of Dutch, did not have any reading disabilities, had normal or corrected-to-normal vision and were right-handed. Hand dominance was assessed with an abridged Dutch version of the Edinburgh Inventory (Oldfield, 1971). This study was approved by the ethical committee of the faculty of Social Sciences of Radboud University. Six participants were excluded from the analyses due to equipment failure and muscular artifacts, leaving a total of 32 participants.

### Materials

A total of 100 Dutch subject–relative (SR) sentences with center-embedded clauses were presented. Of these sentences, 68 were used in Vissers et al. (2010), the other 32 were constructed for this study. For each sentence, a syntactically correct and incorrect version were created, yielding a total of 200 sentences. The incorrect sentences contained subject–verb agreement errors: the verb ending the relative clause did not have the same grammatical number as its subject. The incorrect sentences were derived from the correct sentences by switching the two noun phrases. For example, in the correct sentence ‘De kameel die op de toeristen afliep…’ (The camel who toward the tourists walked[singular]…), the head of the relative clause (‘de kameel’) was switched with the noun phrase in the relative clause (‘de toeristen’) creating the incorrect sentence ‘De toeristen die op de kameel afliep…’ (The tourists who toward the camel walked[singular]…). Because the two noun phrases always differed in number, the switch always yielded a subject–verb agreement error. This way, the correct and incorrect sentences did not differ at the verb critical position. One half of the sentences started with a singular noun phrase, whereas the other half started with a plural noun phrase. Furthermore, the grammatical number of the noun phrases was crossed with sentence grammaticality. The verbs used in the sentences all had a past tense plural inflection that involved the addition of one syllable (e.g., ‘liep’ [walked, 3 singular], versus ‘liepen’ [walked, 3 plural]). In this way, there was maximum discriminability between the plural and singular verb-forms, while holding the length of the verbs constant across conditions.

The two versions of each sentence were counterbalanced across two lists. This means there was no repetition of the experimental sentences within participants. Each list contained 50 SR acceptable and 50 SR unacceptable sentences. 100 filler sentences were added to each list: 25 acceptable SR sentences, 25 unacceptable SR sentences, 25 right-branching sentences (e.g., ‘De taxateur keek naar de schilderijen die veel waard leken’ – ‘The appraiser looked at the paintings that seemed worth a lot’) of which half contained a subject–verb agreement error at the sentence-final verb, and 25 conjunctions (e.g., ‘De huisvrouw kookte voor de kinderen en deed daarna de afwas’ – ‘The housewife cooked for the children and then did the dishes’) of which half contained a subject–verb agreement error at the verb right after the conjunction, yielding a total of 50 acceptable and 50 unacceptable filler sentences. The experimental and filler sentences were mixed in the same pseudo-random order for each of the two lists, with the conditions distributed evenly over lists.

The sentences in both lists were allocated to four blocks. There were two blocks for each task (syntactic and physical). All blocks contained 25 experimental and 25 filler sentences, with the conditions distributed evenly over blocks. In the syntactic task, all words in all sentences were presented in uppercase letters. In the physical task, all words in all experimental sentences were presented in uppercase letters, whereas all filler sentences contained a word in lowercase letters. The physical deviation of the word in lowercase letters was only positioned in the filler sentences to avoid a confound by comparing a single violation in the syntactic task with a double violation in the physical task (i.e., an incorrect verb that was in a deviant letter size) in the experimental sentences. The location of the word in lowercase letters differed per sentence type: for the SR filler sentences, the verb ending the relative clause was in lowercase letters (e.g., ‘DE PINGUIN DIE ONDER DE IJSSCHOTSEN dook BEVOND ZICH OP DE ZUIDPOOL’– ‘THE PENGUIN THAT BELOW THE ICE dived WAS ON THE SOUTH POLE’ [literal translation]), whereas both for the right-branching fillers and the conjunctions the last word of the sentence was printed in lowercase (e.g., ‘DE HUISVROUW KOOKTE VOOR DE KINDEREN EN DEED DAARNA DE afwas’ – ‘THE HOUSEWIFE COOKED FOR THE CHILDREN AND THEN DID THE dishes’). We varied the location of the word in deviant letter size to make sure that participants could not predict where the word in lowercase letters would be located in the sentence.

For both lists, another list was created in which the task blocks were switched, generating a total number of four lists. This way, every sentence was assigned to the syntactic task in one of the lists and to the physical task in another list.

### Procedure

Participants were seated in an enclosed room. A response device with three pushbuttons was set on a table in front of the participant. The sentences were presented in serial visual presentation mode at the center of a PC monitor. Word duration was 345 ms and the stimulus-onset asynchrony (SOA) was 645 ms. Sentence-final words were followed by a full stop. The inter-trial interval was 2 s. Words were presented in black letters on a white background in font size Arial 20 at a viewing distance of ∼1 m. Each sentence was preceded by a 510 ms fixation cross followed by a 500 ms blank screen. Because eye movements distort the EEG recording, participants were trained to make eye movements, e.g., blinks, only after the sentence-final word had disappeared from the screen.

There were two blocks for each task. The experimental session of each task started with a training set of 10 sentences, others than those used in the experiment. Half of the participants started with the syntactic task, the other half started with the physical task.

In the syntactic task participants were instructed to direct their attention to the grammaticality of the sentences. After offset of the sentence-final word, participants had to indicate whether the sentence was syntactically correct (press right button with right index finger) or incorrect (press left button with left index finger).

In the physical task participants were instructed to direct their attention exclusively to the physical features of the sentences. After offset of the sentence-final word participants had to indicate whether the words comprising the sentence were presented in the same font (press right button with right index finger) or not (press left button with left index finger). The maximum response time in both tasks was 3 s, measured from the offset of the sentence-final word.

Immediately before the EEG recording, the mood induction procedure (MIP) was initiated with the first of affective film clips. Between experimental blocks, new film clips were shown. Dependent on the mood condition short film clips were presented from a happy movie or a sad movie. The happy movie fragments were cut from Warner Brothers’ movie Happy Feet; the sad movie fragments were cut from the Universal Pictures’ second World War drama Sophie’s Choice. The film clips showed unambiguous, unipolar emotions and affective situations. Participants were asked to use the situations and emotions depicted in the clips to help them enter the specified mood. The film clips were presented on the same PC monitor used for the presentation of the sentences. The length of the film clips varied between 4.13 and 12.07 min, with a mean length of 7.17 min for the happy mood condition and a mean length of 7.42 min for the sad mood condition. A total of four film clips was presented to the participants. This with the aim to prolong the intended mood during the entire experiment. Mood was manipulated between subjects for two reasons: first, it is difficult to switch on and off a positive vs. negative mood within one single recording session. A possible solution to this would have been to invite the same groups of participants over for a second recording session. This, however, would have resulted in repetition of the critical experimental sentences. Given that language-relevant ERP components like N400 and P600 are sensitive to stimulus repetition (Olichney et al., 2006) and that it takes a long time for stimulus repetition to vanish (Cave, 1997), we preferred not to present the stimulus materials twice.

To assess the effectiveness of the film clips in inducing the intended mood, participants were asked to rate their mood after each movie. The scale ranged from ‘extremely sad’ (-10) to ‘extremely happy’ (+10). In addition, to determine the effectiveness of the instruction to focus attention on syntactic vs. purely physical features of the sentences, participants were asked to fill out an attention rating after each block. They had to indicate how well they could fully direct their attention to the grammatical aspects of the sentence (after the syntactic task blocks) or to the physical aspects of the sentence (after the physical task blocks). The scale ranged from ‘extremely bad’ (-10) to ‘extremely well’ (+10).

### EEG Data Acquisition and Analyses

The electroencephalogram (EEG) was recorded from 26 electrodes mounted in an elastic cap (Acticap system) at standard 10–20 locations. Four electrodes were placed over the midline Fz, Cz, Pz, and Oz. Eleven pairs were placed over the lateral sites F7/F8, F3/F4, Fc5/Fc6, Fc1/Fc2, T7/T8, C3/C4, Cp5/Cp6, Cp1/Cp2, P7/P8, P3/P4, and O1/O2. During recording, the right mastoid served as reference. An electrode was also placed at the left mastoid. The electro-oculogram (EOG) was recorded bipolarly; vertical EOG was recorded by placing an electrode above and below the right eye and the horizontal EOG was recorded by placing two electrodes at the outer left and right canthi. The signals were amplified (time constant = 8 s, bandpass = 0.02–30 Hz), and digitized online at 200 Hz.

Before the analysis, the EEG signals were re-referenced to the mean of the left and right mastoid. EEG and EOG recordings were examined for artifacts and for excessive EOG amplitude (>100 μV) from 100 ms before the onset of the critical verb ending the relative clause to 1 s following its onset. Averages were aligned to a 100-ms baseline preceding the critical verb.

Time-course analyses were conducted to examine the onsets and durations of the ERP effects. To this aim, the mean amplitudes of consecutive time-windows of 100 ms were computed for the different conditions for each participant, beginning at the onset of the critical verb and ending 1 s later. Based on these time course analyses and to increase comparability with previous studies (e.g., Van Herten et al., 2005; Vissers et al., 2010), mean amplitudes in the 600–800 ms time window after critical word onset were used to quantify P600 effects.

To check for early effects of attention, supplementary analyses were performed for the P1 (125–175 ms time window) and N1 (175–225 ms time window) components. The P1 and N1 components are taken to reflect perceptual and attentional processing, respectively (Mangun and Hillyard, 1991; Mangun, 1995). An effect of task for these earlier ERP components would support the view that the task manipulation led to differences in early perceptual and/or attentional processes. Moreover, an interaction including mood and correctness would indicate that the MIP led to differences in early perceptual and/or attentional processes between participants in the happy vs. sad mood condition.

Repeated-measures ANOVA’s were performed to analyze the ERP data for all time windows. The repeated-measures ANOVA’s were conducted separately for the midline sites and for the lateral sites, with correctness (correct vs. incorrect) and task (syntactic vs. physical) as within-subject factors and mood (happy vs. sad) as a between-subject factor. The midline analyses included the additional factor site (Fz, Cz, Pz, Oz). To further explore the scalp distribution of the ERP effects for the lateral sites we used a hemisphere by lateral site (F7/F3/Fc5/Fc1/T7/C3/Cp5/Cp1/P7/P3/O1 vs. F8/F4/Fc6/Fc2/T8/C4/Cp6/Cp2/P8/P4/O2) design. The multivariate approach to repeated measurements was used to avoid problems concerning sphericity (e.g., Vasey and Thayer, 1987). Wilks’ lambda was used to test whether there were differences between the means of the groups of subjects on the (combination of) dependent variables.

To test whether the in the present study reported modulations in P600 amplitude by mood were accompanied by changes in emotional state, correlation analyses were performed. Factors for these correlation analyses were the size of the P600 effect (computed by the difference in amplitude to incorrect and correct verbs) and mean mood rating (computed over mood ratings per subject). To test whether modulations in P600 amplitude were accompanied by changes in the amount of attention a participant directed at the syntactic vs. physical features of the words comprising the sentences, additional correlation analyses were performed. Factors for these analyses were the size of the P600 effect and mean attention rating (computed over attention ratings for the syntactic task and attention ratings for the physical task separately).

## Results

### Mood Induction Procedure

**FIGURE 1:**
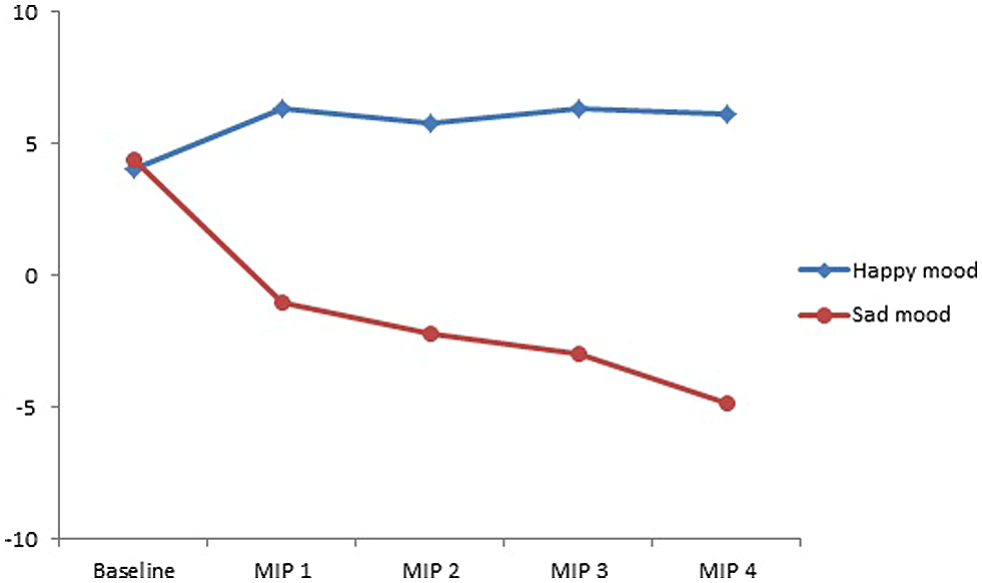
**Mean mood rating scores ranging from -10 (extremely sad) to +10 (extremely happy) for the four film clips comprising the mood induction procedure, separately for the participants assigned to the two mood conditions (happy vs. sad mood condition)**.

As **Figure 1** shows and supported by the statistical analyses reported below, the intended mood was effectively induced by the MIP. That is, participants were significantly happier after watching happy film clips than after watching sad film clips (*p* < 0.001). Likewise, participants were significantly sadder after watching sad film clips than after watching happy film clips (*p* < 0.001). Moreover, participants were significantly happier after watching each of the four happy film clips than they were at the baseline measurement (*ps* < 0.006). Similarly, after watching each of the sad film clips, participants were significantly sadder than they were at the baseline measurement (*ps* < 0.001). There was no difference in mood score between the participants in the happy mood condition (*M* = 4.06, *SD* = 0.53) and the participants in the sad mood condition (*M* = 4.36, *SD* = 0.52) before the film clips were presented [*t*(30) = -0.42, *p* = 0.68].

### Reaction Time and Error Data

The RT and error data of 2 of the 32 participants were removed from the analyses, due to equipment failure. The RT and error data are presented in **Tables 1 and 2** respectively.

**Table 1:** Mean reaction times (ms).

	Syntactic judgment task			Physical judgment task		
	Correct	Incorrect	Difference	Correct	Incorrect	Difference
Happy	688 (241)	603 (207)	-85^∗^	533 (127)	545 (132)	12
Sad	643 (230)	589 (200)	-54^∗^	516 (111)	500 (87)	-16

* p < 0.02.

**Table 2:** Mean error percentages.

	Syntactic judgment task			Physical judgment task		
	Correct	Incorrect	Difference	Correct	Incorrect	Difference
Happy	5.1	7.3	2.2	0.9	0.3	-0.6
Sad	5.1	8.0	2.9	0.6	2.5	1.9


For RT a main effect of task [*F*(1,28) = 12.94, *p* < 0.002] indicated overall longer RTs in the syntactic task than in the physical task. Furthermore, a main effect of correctness [*F*(1,28) = 13.05, *p* < 0.002] reflected overall longer RTs to correct sentences than to incorrect sentences. There was no main effect of mood (*F* < 1) or interaction between mood and task or mood and correctness (*F*s < 1). For RT, a mood by task by correctness interaction [*F*(1,28) = 4.91, *p* < 0.04] reflected differences between conditions as a function of mood. Follow-up analyses revealed that the task by correctness interaction was more pronounced in the happy mood condition [*F*(1,14) = 20.37, *p* < 0.001] than in the sad mood condition [*F*(1,14) = 4.93, *p* < 0.05]. For the happy mood condition, a correctness effect was present in the syntactic task [*F*(1,14) = 19.84, *p* < 0.002] but not in the physical task (*F* < 2). Also, for the sad mood condition, a correctness effect was present only in the syntactic task [*F*(1,14) = 7.02, *p* < 0.02] and not in the physical task (*F* < 1).

For the error data, a main effect of task [*F*(1,28) = 53.00, *p* < 0.001] reflected that participants made more errors in the syntactic task than the physical task. No main effects of correctness or mood (*F*s < 4), or interactions with these factors (*F*s < 2) were found.

### Event-Related Potentials

**FIGURE 2:**
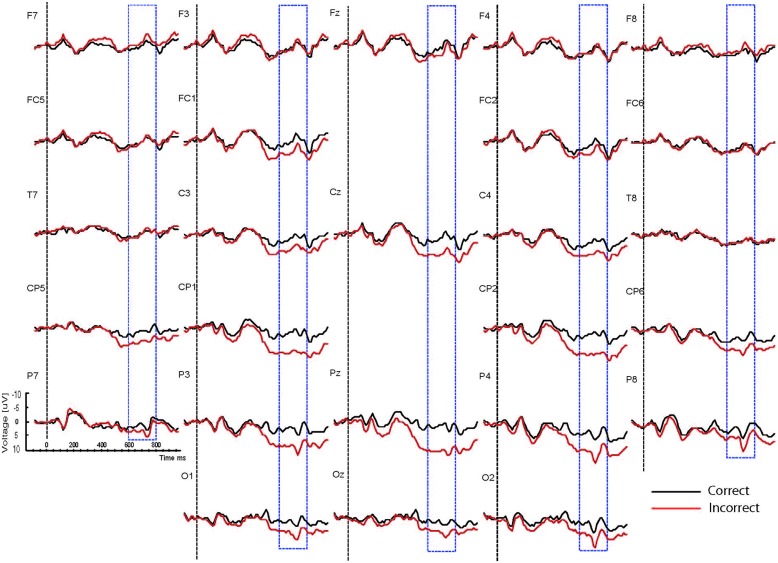
**Grand ERP averages for the happy mood condition in the syntactic judgment task**.

**FIGURE 3:**
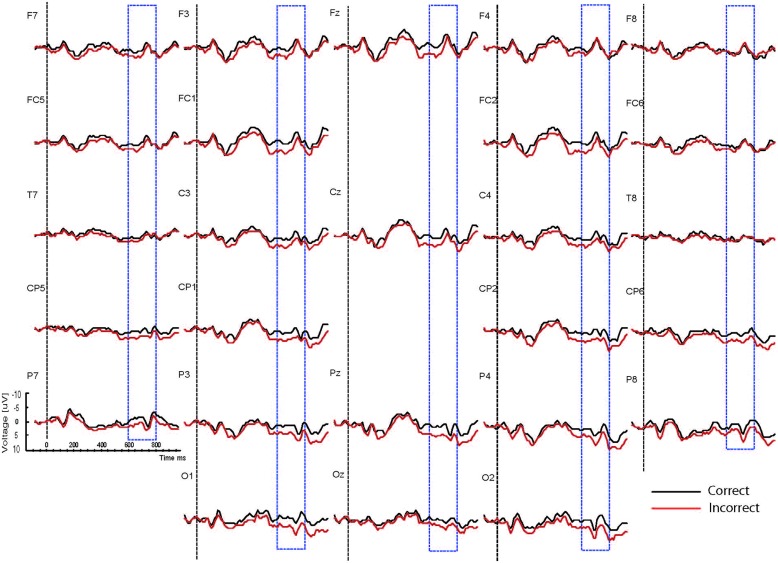
**Grand ERP averages for the happy mood condition in the physical judgment task**.

**FIGURE 4:**
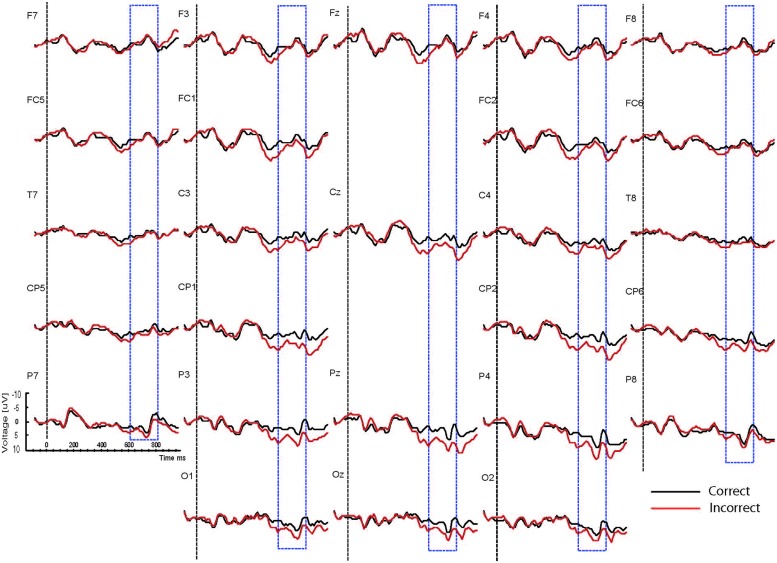
**Grand ERP averages for the sad mood condition in the syntactic judgment task**.

**FIGURE 5:**
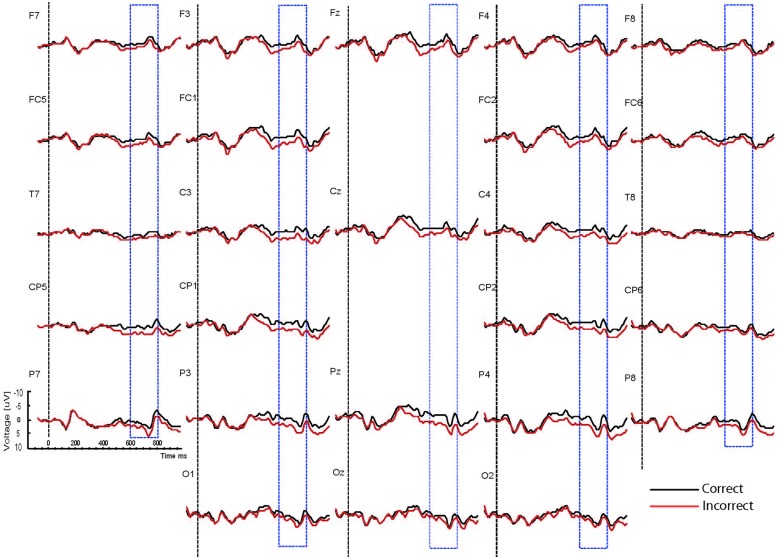
**Grand ERP averages for the sad mood condition in the physical judgment task**.

Based on interactions between mood, task and correctness (see below), the waveforms are presented separately for the two mood conditions (happy vs. sad mood) and the two task conditions (syntactic vs. physical task). The grand mean ERPs to the critical verbs for the happy mood condition for the syntactic task and the physical task are presented in **Figures 2 and 3,**respectively. The grand mean ERPs for the sad mood condition for the syntactic and physical task are presented in **Figure 4 and 5,**respectively.

As the Figures show, the critical verbs elicited an early ERP response that is characteristic for visual stimuli, namely an N1 and a P2, which was preceded by a P1 at occipital sites. These early components were followed by a broad negative wave in the 250–500 ms epoch, peaking at about 400 ms, the N400. The N400 is elicited by each open class word (e.g., Kutas and Van Petten, 1994). The most distinguishing feature of the waveforms was a slow positive shift starting at about 500 ms and extending up to 1000 ms, which was largest at centroposterior sites. This positivity resembles the P600 elicited by syntactic anomalies in terms of its timing and scalp distribution (e.g., Osterhout and Holcomb, 1992; Hagoort et al., 1993). In all conditions, the P600 seemed to be modulated by syntactic correctness, with more positive amplitudes to incorrect verbs than to correct verbs. Visual inspection of the waveforms for the syntactic and the physical task for the two mood conditions suggests (a) that the P600 effect was most prominent for the happy mood condition in the syntactic task and reduced in all other conditions; and (b) the presence of a small N400 effect (i.e., more negative going amplitudes to incorrect verbs than to correct verbs) for the sad mood condition in the syntactic task (see **Figure 4**) and absence of an N400 effect in all the other conditions.

#### P600 Window (600–800 ms)

The percentage of trials excluded from the analyses because of (eye-)movement artifacts was 4.78%. The aim of this study was to examine the combined effects of emotional state and attention on the standard P600 effect to syntactic anomalies. A prerequisite for assessing the effects of both factors on the P600 effect, therefore, is that an effect of correctness is obtained. The time-course analyses revealed correctness effects from 500 up to 1000 ms after critical word onset (*F*s > 9). The largest correctness effects were found in the 600–700 and 700–800 ms time windows (*F*s > 35). Therefore, the 600–800 ms time window measured from critical word onset was used to capture P600 effects.

Main effects of correctness were found for the midline [*F*(1,30) = 50.13, *p* < 0.001] and the lateral sites [*F*(1,30) = 39.75, *p* < 0.001]. These effects reflected that mean amplitudes were overall more positive for incorrect verbs than for correct verbs. No significant main effect of the factor mood was obtained (*F*s < 1). Main effects of task [midline *F*(1,30) = 33.23, *p* < 0.001; lateral *F*(1,30) = 22.70, *p* < 0.001] reflected overall more positive amplitudes in the syntactic task than in the physical task. An interaction between mood, task, correctness and site was obtained for the midline sites [*F*(1,30) = 4.24, *p* < 0.02]. The omnibus ANOVA including all lateral sites did not yield an interaction of mood, task and correctness or interactions of these factors with site and/or hemisphere (*F*s < 3). To test for reliable interactions between mood, task and correctness, region of interest (ROI) analyses were performed for all centroparietal lateral sites that typically yield P600 effects (i.e., C3, CP5, CP1, P7, P3, and O1 for the left hemisphere and C4, CP6, CP2, P8, P4, and O2 for the right hemisphere). The ROI analyses revealed an interaction between mood, task, correctness, hemisphere and site [*F*(1,30) = 3.06, *p* < 0.03]. Based on these interactions separate analyses for the two levels of mood and for the two levels of task were performed for the midline sites and for the lateral centroparietal sites.

##### Happy mood condition: interplay between task and correctness

A main effect of correctness was obtained [midline: *F*(1,15) = 43.27, *p* < 0.001; ROI: *F*(1,15) = 52.51, *p* < 0.001], reflecting a larger mean P600 amplitude to the syntactically incorrect verbs than to the correct verbs. Main effects of task [midline: *F*(1,15) = 11.79, *p* < 0.005; ROI: *F*(1,15) = 15.51, *p* < 0.002] revealed that mean P600 amplitude was larger for the syntactic task than for the physical task. The analyses yielded correctness by site interactions [midline: *F*(1,15) = 30.47, *p* < 0.001; ROI: *F*(1,15) = 22.26, *p* < 0.001] and task by correctness by site interactions [midline: *F*(1,15) = 5.86, *p* < 0.01; ROI: *F*(1,15) = 3.24, *p* < 0.05]. The latter interactions reflected a larger correctness effect in the syntactic task [midline: *F*(1,15) = 32.31, *p* < 0.001; ROI: *F*(1,15) = 48.32, *p* < 0.001] than in the physical task [midline: *F*(1,15) = 7.07, *p* < 0.02; ROI: *F*(1,15) = 10.48, *p* < 0.007]. Furthermore the interaction revealed the presence of a correctness by site interaction in the syntactic task [midline: *F*(1,15) = 30.35, *p* < 0.001; ROI: *F*(1,15) = 21,92, *p* < 0.001], but absence of this interaction in the physical task (*F*s < 2). Follow-up single sites analyses were performed separately for the syntactic task. These analyses revealed P600 effects at centroposterior midline sites (Cz, Pz, and Oz: *p*s < 0.002) and for all centroposterior lateral sites (*p*s < 0.03).

##### Sad mood condition: interplay between task and correctness

Main effects of correctness were obtained [midline: *F*(1,15) = 13.72, *p* < 0.003; ROI: *F*(1,15) = 18.49, *p* < 0.002]. These effects reflected that mean amplitude was larger for the syntactically incorrect verbs than for the correct verbs. Also, a main effect of task [midline: *F*(1,15) = 24.71, *p* < 0.001; ROI: *F*(1,15) = 31.24, *p* < 0.001] reflected overall larger P600 amplitudes for the syntactic task than for the physical task. The analysis yielded task by site interactions [midline: *F*(1,15) = 6.65, *p* < 0.007; ROI: *F*(1,15) = 10.51, *p* < 0.002] and correctness by site interactions [midline: *F*(1,15) = 5.01, *p* < 0.02; ROI: *F*(1,15) = 8.32, *p* < 0.003]. To determine the topography of the P600 effects for the two tasks follow-up analyses were performed for the midline and for centroposterior lateral sites separately for the syntactic and the physical task. For the syntactic task a P600 effect was present at three midline sites (Cz, Pz, and Oz, *p*s < 0.04) and bilateral centroposterior sites (C3, Cp1, P3, O1, Cp2, P4, and O2, *p*s < 0.04). In the physical task a P600 effect was present at posterior midline sites (Pz and Oz, *p*s < 0.04) and bilateral centroposterior sites (C3, Cp5, Cp1, P7, P3, Cp2, P8, and P4, *p*s < 0.04).

#### Comparison of the Size of the P600 Effects Across Conditions

To shed light on the nature of the interaction between mood, task and correctness the size of the P600 effects was compared across conditions. These analyses were carried out on difference scores (incorrect–correct) that directly represented the size of the P600 effect at each site. First we tested for differences in the size of the P600 effects between the two emotional states and second, we tested for differences in the size of the P600 effects between the two tasks.

##### Comparison of the size of the P600 effects as a function of mood

The midline analysis yielded an interaction between mood, task and site [*F*(1,30) = 4.85, *p* < 0.01]. The three-way interaction indicated the presence of a mood by site interaction in the syntactic task [*F*(1,30) = 6.79, *p* < 0.002] and absence of this interaction in the physical task (*F* < 1). Follow-up analyses revealed that in the syntactic task for Pz the P600 effect was significantly larger for the happy mood condition than for the sad mood condition [*t*(30) = 2.94, *p* < 0.01].

The ROI analysis yielded a trend toward an interaction between mood, task, hemisphere and site [*F*(1,30) = 2,36, *p* = 0.07]. This trend is probably caused by larger P600 effects in the syntactic task for the happy mood condition than for the sad mood condition at centroparietal sites (see **Figure 5**). In line with this the difference scores for the centroposterior sites disclosed that in the syntactic task the P600 effect was significantly larger for the happy than the sad mood condition at two lateral sites (Cp1 and P3; *p*s < 0.05).

**FIGURE 6:**
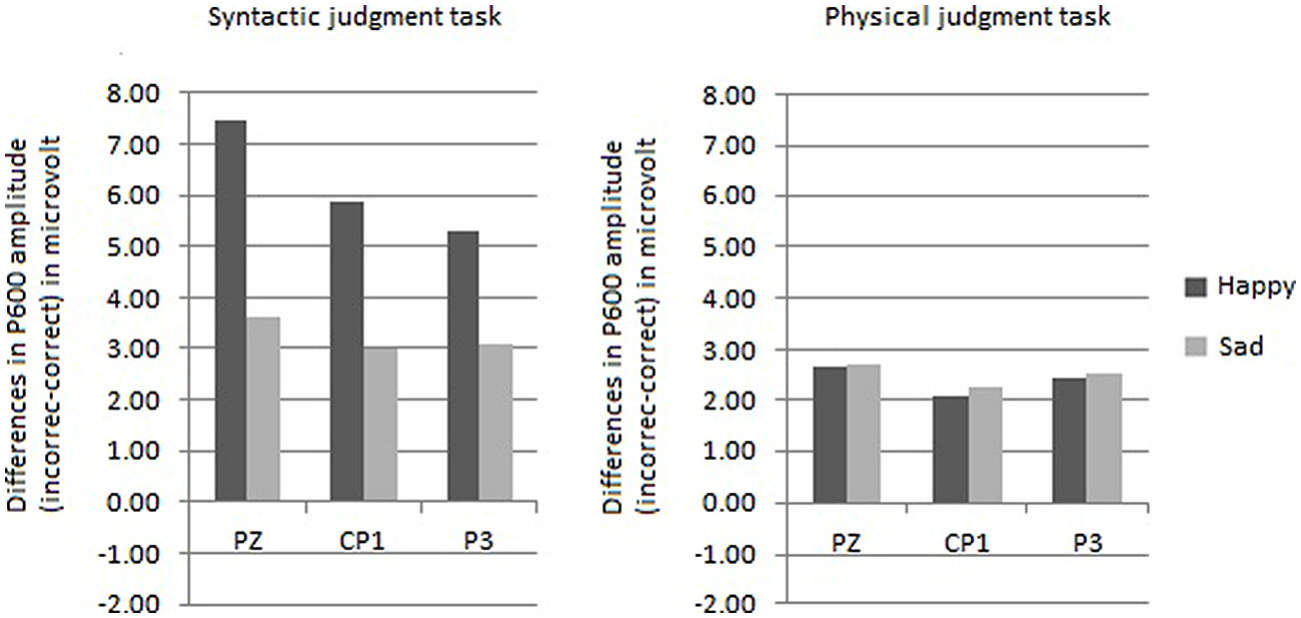
**Comparison of the mean P600 amplitudes (incorrect–correct) for the happy and sad mood condition, for the syntactic judgment task (on the left side) and for the physical judgment task (on the right side) separately**.

##### Comparison of the size of the P600 effects as a function of task

**FIGURE 7:**
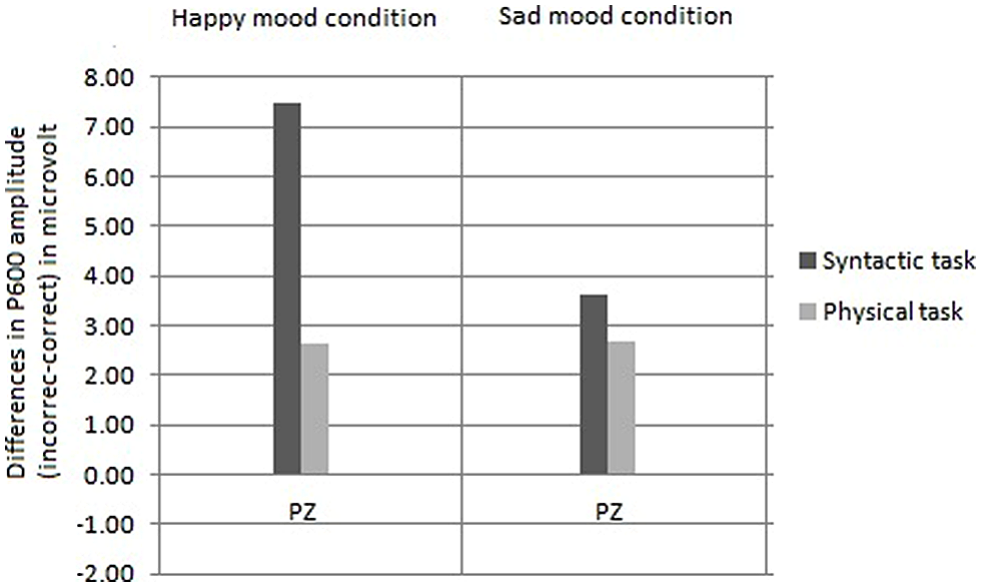
**Comparison of the mean P600 amplitudes (incorrect–correct) for the syntactic and physical task, for the happy mood condition (on the left side) and for the sad mood condition (on the right side) separately**.

Based on the interaction between mood, task and site [*F*(1,30) = 4.85, *p* < 0.01] reported above for the midline the analyses were performed separately for the two levels of mood. In the happy mood condition, an interaction between task and site was obtained [*F*(1,15) = 5.86, *p* < 0.01]. Follow-up analyses disclosed that the P600 effect at Pz was significantly larger in the syntactic task than in the physical task [*t*(30) = 3.10, *p* < 0.008; see **Figure 7**]. In contrast, in the sad mood condition, no task by site interaction was obtained (*F* < 2).

#### Correlation Analyses

To test whether modulations in P600 amplitude in the two moods conditions are accompanied by changes in emotional state, Pearson correlations were calculated between the size of the P600 effect and the mean mood rating (computed over four mood ratings per participant). The size of the P600 effect was computed by subtracting P600 amplitude to the syntactically correct verbs from that to the syntactically incorrect verb; this difference score was computed for the centroparietal midline electrodes (Cz and Pz) and for the subset of lateral centroparietal electrodes (Cp5, Cp1, P3, Cp6, Cp2, and P4) showing the strongest P600 effects. These analyses revealed significant correlations between the size of the P600 effect in the syntactic task and the mood ratings for both centroposterior midline electrodes: Cz (*p* < 0.05) and Pz (*p* < 0.001) and for five of the centroposterior lateral electrodes: Cp5, Cp1, Cp2, P3, and P4 (*p*s < 0.02). These correlations indicated that the happier the mood, the larger the P600 effect and likewise, the sadder the mood, the smaller the P600 effect. With correlations ranging from 0.35 up to 0.60, at least 12% up to 36% of the variation in size of P600 effect is accompanied by variations in emotional state.

To test whether modulations in P600 amplitude in the two mood conditions are accompanied by changes in attention, Pearson correlations were calculated between the size of the P600 effect and the mean attention rating (computed over four attention ratings per participant) as factors. Correlation analyses collapsed across the two levels of mood revealed a correlation between the size of the P600 effect in the syntactic task and the attention ratings for one occipital site (Oz: *p* < 0.02). This correlation indicated that the more attention is paid to the syntactic features, the larger the P600 effect at Oz and likewise, the less attention is paid to the syntactic features of the stimuli, the smaller the P600 effect. With a correlation of 0.42, 18% of the variation in size of the P600 effect is accompanied by variations in the amount of attention paid to the syntactic structure of the sentences.

### Early Attentional Factors

To check for early effects of attention, supplementary analyses were performed for the P1 (125–175 ms time window) and N1 (175–225 ms time window) components.

#### P1

For the P1 component, no main effects of correctness were present for the midline sites or for the lateral sites (*F*s < 2). For the midline sites, a correctness by mood interaction was obtained [*F*(1,30) = 5.42, *p* < 0.03], reflecting that a correctness effect (more positive amplitudes to incorrect than correct verbs) was present for the happy mood condition [*F*(1,15) = 5.60, *p* < 0.04], but not for the sad mood condition (*F* < 1). For the lateral sites, a three-way interaction between mood, correctness and site was obtained [*F*(1,30) = 3.20, *p* < 0.02]. The interaction reflected that a correctness by site interaction was present for the happy mood condition [*F*(1,15) = 4.45, *p* < 0.05], but not for the sad mood condition (*F* < 1). The correctness by site interaction as found for the happy mood condition reflected that a correctness effect was present at a few centroposterior, posterior and occipital sites.

#### N1

For the N1 component, no main effects of correctness were found for the midline and lateral sites (*F*s < 2). Main effects of task were present both for the midline sites [*F*(1,30) = 11.61, *p* < 0.003] and the lateral sites [*F*(1,30) = 7.45, *p* < 0.02]. These effects reflected more negative amplitudes for the syntactic task than for the physical task. For the midline, a three-way interaction between mood, task and correctness [*F*(1,30) = 5.14, *p* < 0.04] was obtained, reflecting the presence of a two-way interaction between task and correctness for the sad mood condition [*F*(1,15) = 8.24, *p* < 0.02], but not for the happy mood condition (*F* < 1). The task by correctness interaction for the sad mood condition disclosed that the correctness effect was a bit larger, though not significant, in the syntactic task (*F* < 5) than in the physical task (*F* < 4).

## Discussion

In several fields of psychology it has been shown that a person’s emotional state influences the way in which information is processed (see for a review: Clore and Huntsinger, 2007). For instance, it has been shown that a positive compared to a neutral or negative mood facilitates creative problem-solving (e.g., Greene and Noice, 1988), stereotyping (e.g., Fiedler and Walther, 2004) and recalling materials from memory (e.g., Isen et al., 1978). Based on the classic view that language processing occurs in isolation from other cognitive systems, like perception, motor action, and emotion, only recently the interplay between emotion and language has been investigated. The few ERP studies that explored the effects of mood on semantics revealed that mood affects the processing of word meaning as tapped by N400 (Federmeier et al., 2001; Chwilla et al., 2011; Egidi and Nusbaum, 2012; Pinheiro et al., 2013). The reported interactions between mood and semantic processing support interactive theories of language (e.g., Glenberg, 1997; Barsalou, 1999) and present a challenge for modular theories of language comprehension (Fodor, 1983). The effect of emotional state on syntactic processing is more controversial, one study reported a mood-related modulation in syntactic processing as reflected by P600 (Vissers et al., 2010), while two other studies did not observe an effect (Jiménez-Ortega et al., 2012; Van Berkum et al., 2013). While the absence of an effect of emotional state on syntax seems to fit well with the view that syntactic processing is of a modular nature, the finding of a mood by syntax interaction calls this view into question.

The main goals of the present article were as follows: the first goal was to clarify whether and if so how emotional state affects syntactic processing. The second goal was to shed light on the underlying mechanisms by separating possible effects of mood from those of attention on syntactic processing. To these aims, we manipulated attention next to the emotional state of the participants and investigated the joint effects of these two factors on the processing of syntactic anomalies. Different emotional states (happy mood vs. sad mood) were induced and prolonged by presenting film clips before and between task blocks. Attention was manipulated by task demands. Participants were asked to exclusively pay attention to the syntactic well-formedness of the sentences (syntactic task), or to purely physical features of the words of the sentences (physical task).

A necessary condition for the investigation of the relationship between mood, attention and the processing of syntactic anomalies as reflected by P600, is that the mood induction was successful. The behavioral results reveal that this was the case. As indicated by the analyses of the mood ratings, the intended mood was successfully induced. Participants were in a significantly happier mood after watching the happy film clips and they were in a significantly sadder mood after watching the sad film clips (see **Figure 4**).

**FIGURE 8:**
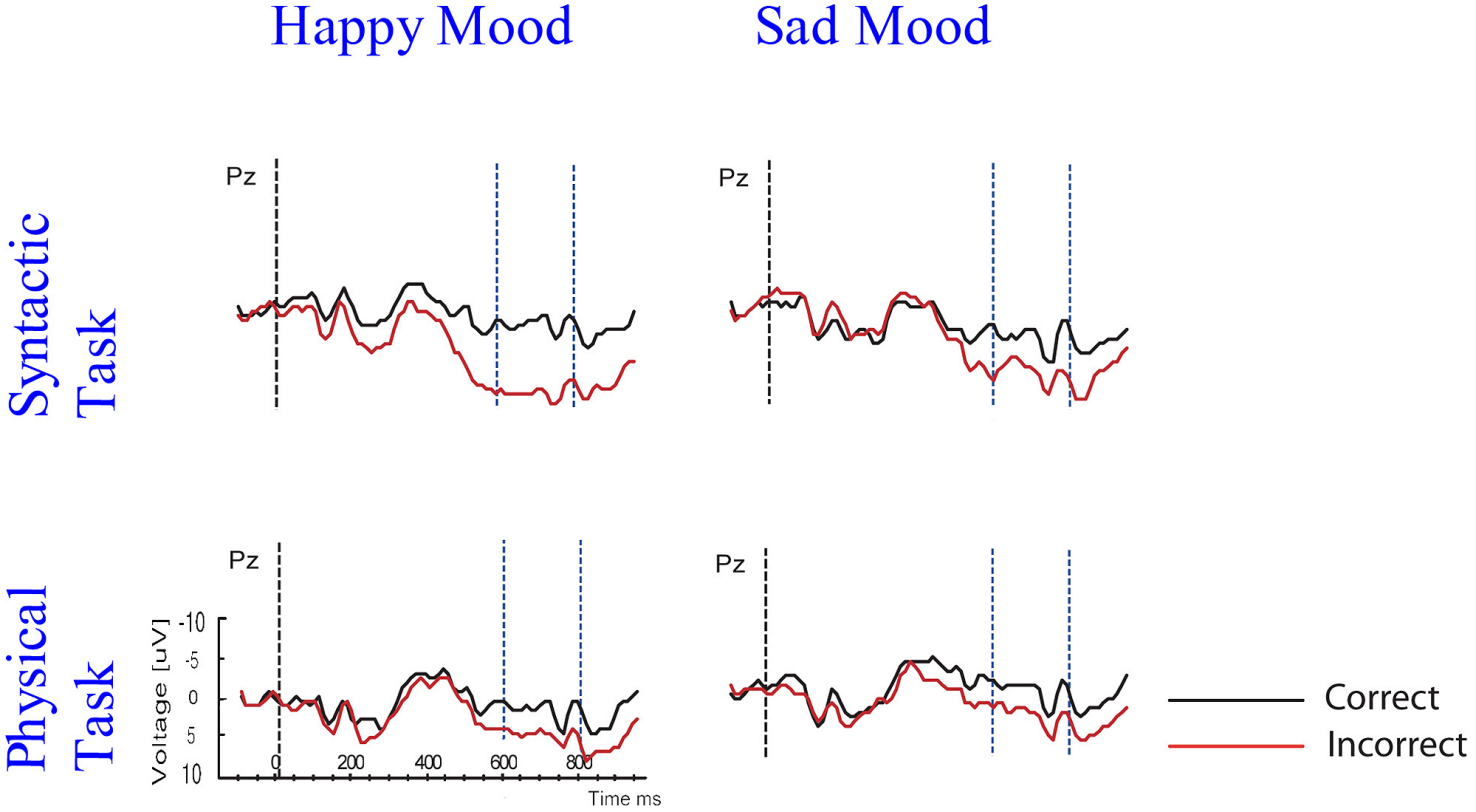
**Grand ERP averages at PZ for both mood and task conditions**.

With these data in hand we can address the questions whether emotional state affects the P600 effect and whether attention influences the mood-related modulation of P600. The main ERP results were as follows: as predicted a standard P600 effect was elicited by subject–verb agreement errors, across mood and task conditions. More importantly, interactions between mood, attention, and correctness were obtained for the midline and the lateral centroposterior sites that typically show largest P600 effects to syntactic violations. The interactions reflected a modulation of P600 as a function of both emotional state and attention (see **Figure 8**).

### Influence of Emotion and Attention on P600

Let us first describe the influence of emotional state on the P600 effect. Emotional state only affected P600 in the syntactic task and not in the physical task. In particular, a larger P600 effect was found in the happy than in the sad mood condition. This was supported by the results of the difference scores analyses and correlation analyses. The fact that emotional state only affected P600 in the syntactic task – and not in the physical task – seems to indicate that a necessary condition for effects of emotional state on syntactic processing to occur is that (some) attention is directed at the syntactic level. Apparently, the effects of mood on syntactic processing are influenced by attentional demands.

We will now examine the influence of attention on the syntactic correctness effect on P600. The focus of attention only had an impact on the P600 in the happy mood condition and not in the sad mood condition. Specifically, for happy mood a reduction in P600 effect was found for the physical compared to the syntactic task. This was supported by the results of the difference scores analyses and correlation analyses. As stated above a task-related modulation of the P600 effect was only present in the happy mood condition, and not in the sad mood condition. In other words, while a standard P600 effect occurred in the syntactic and the physical task, directing attention at syntactic vs. physical features only affected syntactic processing – as reflected by changes in P600 amplitude – when participants were in a happy mood.

Let us summarize the main results in relation to the goals of the present article. The first goal of this study was to clarify whether emotional state has an effect on syntactic processing. As aforementioned, the findings in the literature are controversial. While Vissers et al. (2010) reported a mood-related modulation of the P600 effect to subject–verb agreement errors, Jiménez-Ortega et al. (2012) and Van Berkum et al. (2013) did not find an effect of mood on the processing of syntactic anomalies. The in the present study reported mood-related modulation of the P600 effect to syntactic anomalies is consistent with the results of Vissers and colleagues. Therefore, one major finding of this article is that we replicated an immediate effect of emotional state on the processing of syntactic anomalies. From this we conclude that syntactic processes – opposite to what has been proposed (Van Berkum et al., 2013) – are affected by changes in emotional state. This means that the effects of emotional state on language comprehension are not limited to semantic processing but also involve syntactic processing.

The second goal of this study was to separate effects of mood on syntactic processing from those of attention. Phrased differently, what role do more general factors like attention play in the mood-related modulation of the syntactic P600 effect? A comparison of the present P600 results with those of Vissers et al. (2010) helps to answer this question. Directing the attention of the participants in the present study modulated the mood-related P600 effect. In particular, directing attention to syntactic features diminished the effect of mood on the P600 effect compared to the previous study, in which participants read for comprehension. In the latter study the P600 effect was strongly reduced in the sad mood condition (i.e., only present at two lateral sites). In contrast, in the present study a broadly distributed P600 effect was found in the syntactic task for the sad mood condition (i.e., present at three midline and eight centroparietal lateral sites). The P600 results reveal that directing attention to the syntactic level reduced the immediate impact of emotional state on the processing of syntactic anomalies. However, most important for the present purposes, the effect of emotional state was not abolished as evident from that, as **Figure 6** illustrates, the P600 effect was smaller for the sad mood than for the happy mood condition. Additionally, the focus of attention modulated the effect of emotional state on the processing of syntactic anomalies, in that no effect of emotional state was observed in the physical task. From this we draw the conclusion that attention plays a modulating role in the effect of emotional state on syntactic processing. As pointed out above, emotional state also modulated the effect of attention on the processing of syntactic anomalies. This is apparent from that the task manipulation only affected the P600 effect in the happy mood condition and not in the sad mood condition. To conclude, the three-way interaction indicates a reciprocal influence of attention on emotion and of emotion on attention. Clearly future work is needed to further our understanding about the interplay of attention and emotion in language comprehension.

The fact that more general non-linguistic factors like emotion and attention impact language processing, in particular syntactic processing, supports interactive theories of language (e.g., McDonald et al., 1994; Trueswell and Tanenhaus, 1994; Barsalou, 2008) and challenges modular views according to which language processes operate in isolation from other cognitive and linguistic sub-systems (see e.g., Forster, 1979; Fodor, 1983). The present ERP data accord well with the results of fMRI studies that have indicated that emotion and cognition (memory, attention, language) strongly interact in the brain (e.g., Mitchell and Phillips, 2007; Pessoa, 2008). Brain regions that are often associated with cognitive processing, such as the lateral prefrontal cortex, have now been shown to be strongly involved in both affective and cognitive function (i.e., Gray et al., 2002). Moreover, these fMRI results have been taken to indicate that brain regions previously viewed as purely affective, like the amygdala, hypothalamus and anterior cingulate cortex, are among the most highly connected regions of the brain and might function as important connectivity hubs (Pessoa, 2008). Where these fMRI studies disclose that the neural correlates of emotion and cognition should be viewed as interactive, the present ERP results reveal an immediate interaction of emotional state and attention on the processing of syntactic anomalies. Before turning to possible mechanisms that give rise to emotion by language interactions for P600 we will now look at effects of attention and mood on early ERP effects.

### Early ERP Effects of Attention and/or Emotional State on Syntactic Processing

The attention manipulation gave rise to early ERP effects. Main effects of task were present for N1, reflecting more negative amplitudes for the syntactic task than for the physical task. It has been well established that directing attention to some aspect of the environment leads to an enhancement of N1 amplitude (Hillyard and Anllo-Vento, 1998). In line with this, the larger N1 amplitude for the syntactic task is taken to indicate that participants paid more attention to the critical verbs in the syntactic task than in the physical task. This difference could be influenced by the task manipulation itself, because participants were asked to focus their attention on the syntactic features of the sentences in the syntactic task and on the physical features in the physical task. Note that the fact that only in the syntactic task the critical verb was task-relevant could also play a role. In order to avoid a double violation (syntactic violation plus different letter size), in the physical task only words occurring in the filler sentences were task-relevant (i.e., printed in a smaller font; see below).

The mood manipulation gave rise to early effects in the P1 window. In particular, interactions between mood and correctness were found, reflecting the presence of a correctness effect in the happy mood condition and absence of a correctness effect in the sad mood condition. Early effects of emotional meaning on P1 have been reported before (see Scott et al., 2009; Bayer et al., 2012). These effects were taken to indicate that emotion affects early stages of processing. The present results may be taken to suggest that the early emotion effects are not restricted to emotional language but generalize to neutral language, as emotionally neutral words were used in the present study. However, future studies on the effects of emotional state under attended and unattended conditions are required to understand the functional significance of these early effects of attention and emotion on the N1 and P1 component, respectively.

### Possible Mechanism(s) Behind the Emotion by Language Interactions

What could be the mechanism(s) underlying the effect of emotional state on syntactic language processing? In the introduction three possible explanations for the mood-related P600 modulation have been presented. According to one scenario emotional state influences syntactic processing. Several syntactic manipulations have been shown to elicit a P600. The P600 effect has been taken to reflect processes of syntactic reanalysis (e.g., Friederici, 1995; see for a more general reanalysis account of the P600: Kolk and Chwilla, 2007), syntactic processing *per se* (Hagoort et al., 1993), or syntactic integration difficulty (Kaan et al., 2000). On a syntactic account, the decrease in P600 effect in the sad compared to the happy mood condition could reflect reduced syntactic processing. Alternatively, happy mood could lead to an enhancement of syntactic processing (for a further discussion of the syntactic account, see below). The role of syntactic processing in bringing about effects of emotional state on sentence processing could be explored in future studies by varying syntactic factors, for instance syntactic complexity, alongside emotional state.

According to a second scenario mood might influence syntactic processing via more general factors such as attention or motivation (Vissers et al., 2010, 2013; Chwilla et al., 2011). Regarding a possible mediating role of attention, this is the first study that directly assessed the joint effects of attention and emotional state on the processing of syntactic anomalies. The present P600 results show that attention plays a modulating role in the mood-related P600 effect: directing attention to the syntactic features of the sentence reduced the influence of emotional state on syntactic processing. Importantly, however, it did not abolish the effect – that is, the P600 effect was smaller for the sad mood than for the happy mood condition. From this we can conclude that there is a genuine effect of emotional state on the syntactic P600 effect that cannot be accounted for by attention. Interpreting the interaction between mood and syntactic processing in terms of attention and/or motivation lines up with a language processing model (MRC hypothesis) proposed by Brouwer et al. (2012; see Brouwer and Hoeks, 2013, for an extended discussion of this hypothesis). According to these authors, P600 reflects word-by-word construction or updating mental representations of what is being read. P600s to syntactic anomalies can thus be taken to reflect increased effort in integrating the critical word with its prior context to form a coherent representation. On this account, the broadly distributed P600 effect for the happy mood condition could reflect a strong effort to syntactically integrate words, while the reduction in P600 effect for the sad mood condition could reflect a reduced integration effort. Put shortly, happy participants could either pay more attention to syntactic features or could be more highly motivated to process syntactic information, which could be reflected by an increase in P600.

Consistent with an explanation in terms of motivation, Van Berkum et al. (2013) found an influence of emotional state on the processing of verb-based expectancies. In particular, they investigated the effect of mood on the anticipation of referents during discourse comprehension. They presented participants with sentences in which a pronoun was highly expected (“Sarah feared Joe because he…”) vs. sentences in which a pronoun was not highly expected (“Joe feared Sarah because he…”). The authors found that mood affected referential anticipation. People in a happy mood did anticipate referential information, whereas people in a sad mood did not anticipate information about a specific person. Relevant for the present discussion, as stated above in the same study Van Berkum et al. (2013) did not find an effect of mood on the processing of syntactic agreement violations. They proposed a bio-energetic explanation to account for the presence of a mood effect on the processing of verb-based expectancies vs. an absence of a mood effect on syntactic parsing. According to this explanation, mood has an influence on how willing people are to invest in costly, exploratory behavior, such as referential anticipation. On this account, people in a sad mood would be less motivated to invest in exploratory processing than people in a happy mood. For people in a sad mood, the benefits of exploratory processing would not outweigh the perceived bioenergetics costs. The authors argue that referential anticipation requires greater mental effort than syntactic parsing. In this way, they explain that emotional state does have an influence on referential anticipation but not on syntactic processing.

A third scenario is that a person’s emotional state influences the use of heuristics. On this account, the decrease in P600 effect for the sad mood compared to happy mood reflects that people in a happy mood rely more on heuristic processing than people in a sad mood. Heuristics are very effective in extracting meaning and allow people to quickly solve problems and make judgments (Ferreira, 2003). The proposal that mood influences the use of heuristics fits well with the notion of mood-dependent processing styles. In happy mood, people are inclined to rely more on heuristic processing than people in a sad mood (Clore and Huntsinger, 2007). When using heuristics, people base their interpretation on a “good-enough” interpretation of the information. In other words, people do not take all information into account, but settle for a representation of the input that fits with their expectation based on their world knowledge. Ferreira (2003) and Ferreira and Patson (2007) have claimed that current models of language are missing an architectural component that explains cases in which heuristic processing is engaged. Relevant in this context, it has been shown that P600 is sensitive to heuristic factors (e.g., Coulson et al., 1998a; Vissers et al., 2007). This is indicated by the fact that semantic reversal anomalies like “The cat that fled from the mice” elicit a P600 effect compared to the based on world knowledge expected event “The mice that fled from the cat” (Kolk et al., 2003; Kim and Osterhout, 2005; Van Herten et al., 2005). Given that semantic reversals are syntactically unambiguous, they allow an assessment of the contribution of heuristic processing to the mood-related P600 modulation. Therefore, Vissers et al. (2013) investigated the effect of emotional state on the processing of semantic reversal anomalies. The main result was that for P600, a mood by semantic plausibility interaction was obtained. The interaction reflected a widely distributed P600 effect for the happy mood condition vs. absence of a P600 effect for the sad mood condition. Based on the fact that semantic reversal anomalies are syntactically unambiguous, the P600 modulation by mood cannot be explained by syntactic factors (Scenario 1). A direct statistical comparison of the emotion effect on the two kinds of anomaly revealed that the effect of mood, as reflected by modulations in P600, on the processing of semantic reversal anomalies was similar to the effect of mood on the processing of subject–verb agreement errors. Taken together, the results of Vissers et al. (2007, 2013) support the claim that heuristics play an important role in the mood by language interactions. Based on the assumption that language users expect to read syntactically correct sentences (Coulson et al., 1998a,b; Vissers et al., 2010, 2013) the in the present article reported increase in P600 effect in happy mood thus could reflect an increased use of heuristics, whereas the reduction of P600 effect in sad mood could reflect a reduced use of heuristics. Nota bene: that the mood manipulation only led to a reduction in P600 for sad as compared to happy mood when attention was directed at syntactic features (and not in the physical task) seems to fit well with a heuristic account of the interplay between mood and syntactic processing. After all, if people expect sentences to be syntactically correct, heuristics mainly play a role in the syntactic condition and not in the physical condition.

### Caveats

The above claims can only be made if it can be shown that the observed differences in ERP pattern between emotional states and task conditions could not be attributed to other factors.

One point to discuss regarding the design of the present study, is that to avoid a double violation on the critical verb (i.e., an incorrect verb that was in a deviant letter size), the change in letter size (lowercase instead of uppercase) was only present in the filler sentences. The reason for this was that physically deviant words have been shown to elicit a P3b, a positive component with an average latency of 300 ms (see e.g., Kutas and Hillyard, 1980; Donchin, 1981). Relevant in this context, Arbel et al. (2011) investigated the influence of a double violation on the N400 and P3b. For words that were both semantically and physically deviant, a smaller P3b and a larger N400 was elicited than for words that only contained a physical violation or a semantic violation, respectively. Arbel et al. (2011) propose that this modulation of P3b and N400 effects reflected that either attentional resources were allocated to the semantic rather than the physical characteristics of the stimuli, or that fewer resources were allocated for the processing of the physical characteristics because these were task-irrelevant. In the present study, we ruled out that a physical deviation on the critical verb would result in extra attention for the syntactic correctness of the sentence. By manipulating the letter size only in the filler sentences, we made sure that the physical task manipulation indeed resulted in less attention for the syntactic structure of the sentence. This, however, leads to a difference in the present study between tasks. While in the syntactic task the critical verb was task-relevant, the same set of critical words in the physical task was not task-relevant. This likely has affected the P600 in the present study across tasks, given that the P600, like the P3b, is sensitive to task demands with larger P600 amplitudes to task-relevant stimuli (e.g., Donchin, 1981). The in the present study observed main effect of task, therefore, could partly be due to these differences in task relevance of the critical verb in the syntactic vs. physical task. However, most important for the present purposes, this difference between tasks does not affect the within-task comparison of the ERP patterns across the two mood conditions.

Another consideration is that task difficulty was not controlled across tasks. This resulted in differences in overall RTs and errors between the syntactic and the physical task. There was an increase in both RTs and errors in the syntactic task as compared to the physical task. The behavioral data show that task difficulty was not matched between tasks. Could this difference in task difficulty – as reflected by differences in RTs – explain the present ERP results? It is important to point out that a different pattern was observed for the P600- and the RT-measure. While P600 amplitude across mood conditions was reduced for correct as compared to syntactically incorrect verbs, overall RTs were longer to correct than incorrect verbs. This speaks against an explanation of the present P600 pattern in terms of RT. A similar increase in RT for correct as compared to incorrect verbs has been reported by Kolk et al. (2003) and Vissers et al. (2007). In line with these authors we propose that the difference in RTs in the present study could be explained by the fact that in the case of the incorrect sentences, participants already knew at the critical verb that the sentences were incorrect. In contrast, in the case of the correct sentences they had to wait until they had read the last word of the sentence before they could know for sure that the sentence was correct. Mood by task by syntactic correctness interactions were found for RT and P600. Closer inspection shows that the three-way interactions reflect different patterns for the two measures. For RT the interaction indicates that the correctness effect (an increase in RT for correct compared to incorrect sentences) in the syntactic task was larger for happy mood than for sad mood. Note that for the physical task, no correctness effect was found for RT, neither for the happy mood nor for the sad mood condition. In contrast, for P600 the three-way interaction reveals another picture. Importantly, for P600 correctness effects (smaller P600 amplitude for syntactically correct than incorrect verbs) were present both in the syntactic task and in the physical task as well as across mood conditions. Thus although mood by task by correctness interactions were obtained for RT and P600 the underlying data patterns are different. Last but not least we would like to point at another essential difference between behavioral and electrophysiological measures. ERPs track the language processes of interest online, time-locked to the critical verb. In contrast, the behavioral data are measured oﬄine after the sentence-final word. The mean difference in time between the onset of the critical verb and the offset of the sentence-final word was 1666 ms. The behavioral response, thus, followed the online ERP response to the critical verb by more than 1.5 s. Based on this difference in the timing of the behavioral and ERP response – and most importantly based on the arguments presented above – we consider it highly unlikely that the in the present study observed differences in ERPs between conditions can be attributed in a simple way to differences in RTs.

## Conclusion

To our knowledge this is the first ERP study that demonstrates that emotional state and attention have interactive effects on language comprehension, in particular on the processing of syntactic anomalies. The present ERP results demonstrate that emotional state modulates the P600 effect to syntactic agreement errors. The major novel finding from the present study is that more general factors like attention play a role in the mood-related modulation of the processing of syntactic anomalies, as reflected by P600. Directing attention to the syntactic level reduced the impact of emotional state on the processing of syntactic anomalies. However, the effect of emotional state was not abolished as evident from the fact that, as in the Vissers et al. (2010) study, the P600 effect was smaller for the sad mood as compared to the happy mood condition. Also, emotional state modulated the effect that attention has on language processing. This is indicated by the fact that the task manipulation only affected the syntactic P600 effect when participants were in a happy mood and not when they were in a sad mood. That emotion and attention interact with syntactic processing supports interactive views of language and further challenges modular theories of language comprehension.

Exploration of the relationship between emotion, attention and processes of language comprehension is still in its infancy. The challenge for future studies is to shed light on the different mechanisms that mediate the influence of emotional state and focus of attention on syntactic processing. Future studies will have to take into account that attention can play a modulating role in the interplay between language and emotion.

## Conflict of Interest Statement

The authors declare that the research was conducted in the absence of any commercial or financial relationships that could be construed as a potential conflict of interest.
